# Microbiome Dysbiosis: A Pathological Mechanism at the Intersection of Obesity and Glaucoma

**DOI:** 10.3390/ijms24021166

**Published:** 2023-01-06

**Authors:** Salvatore Pezzino, Maria Sofia, Luigi Piero Greco, Giorgia Litrico, Giulia Filippello, Iacopo Sarvà, Gaetano La Greca, Saverio Latteri

**Affiliations:** 1 Department of Surgical Sciences and Advanced Technologies “G. F. Ingrassia”, Cannizzaro Hospital, University of Catania, 95126 Catania, Italy; 2 Complex Operative Unit of Ophtalmology, Cannizzaro Hospital, University of Catania, 95126 Catania, Italy

**Keywords:** overweight, ocular disorders, glaucoma, intraocular pressure, gut microbiome, resident brain–ocular microbiome, inflammation, therapies, probiotics

## Abstract

The rate at which obesity is becoming an epidemic in many countries is alarming. Obese individuals have a high risk of developing elevated intraocular pressure and glaucoma. Additionally, glaucoma is a disease of epidemic proportions. It is characterized by neurodegeneration and neuroinflammation with optic neuropathy and the death of retinal ganglion cells (RGC). On the other hand, there is growing interest in microbiome dysbiosis, particularly in the gut, which has been widely acknowledged to play a prominent role in the etiology of metabolic illnesses such as obesity. Recently, studies have begun to highlight the fact that microbiome dysbiosis could play a critical role in the onset and progression of several neurodegenerative diseases, as well as in the development and progression of several ocular disorders. In obese individuals, gut microbiome dysbiosis can induce endotoxemia and systemic inflammation by causing intestinal barrier malfunction. As a result, bacteria and their metabolites could be delivered via the bloodstream or mesenteric lymphatic vessels to ocular regions at the level of the retina and optic nerve, causing tissue degeneration and neuroinflammation. Nowadays, there is preliminary evidence for the existence of brain and intraocular microbiomes. The altered microbiome of the gut could perturb the resident brain–ocular microbiome ecosystem which, in turn, could exacerbate the local inflammation. All these processes, finally, could lead to the death of RGC and neurodegeneration. The purpose of this literature review is to explore the recent evidence on the role of gut microbiome dysbiosis and related inflammation as common mechanisms underlying obesity and glaucoma.

## 1. Introduction

The prevalence of obesity has nearly tripled in the last thirty years, largely as a result of people becoming less active and eating unhealthier diets [1]. Obesity affects people of all ages, races, and socioeconomic backgrounds [1,2]. Obesity has far-reaching consequences, and it is well established that it negatively affects the cardiovascular and metabolic systems [3,4]. A wide spectrum of harmful outcomes is associated with obesity [5]; coronary heart disease, type 2 diabetes mellitus, high blood pressure, stroke, abnormal lipid profiles, osteoarthritis, and sleep apnea are only some of the diseases that include obesity as a risk factor [5,6]. In addition, there is evidence linking obesity to a slew of malignancies [5,7]. On the other hand, the possible effects of obesity on the development of eye diseases are less thoroughly studied. A clinical study has shown a detrimental connection between obesity and visual acuity, but the ocular circumstances underlying this association and its consequences are not well understood [8]. Among several eye diseases, obesity has been linked to glaucoma [9,10], which is another disease of epidemic proportions [11]. Globally, it was predicted that the number of glaucomatous patients is expected to rise to around 110 million by 2040 [11]. Glaucoma is characterized by permanent damage to the optic nerve, which can lead to blindness, and it is the primary worldwide cause of irreversible blindness [11,12]. The advancement of the disease is irreversible but can be slowed by treatment; consequently, it is essential to identify risk factors connected with the condition to facilitate earlier discovery [13].

There is growing interest in microbiome dysbiosis, particularly in the gut, which has been widely acknowledged to play a role in the etiology of metabolic illnesses such as obesity [14,15] and chronic liver diseases [15,16,17]. On the other hand, there is increasing evidence that microbiome dysbiosis plays a critical role in the onset and progression of several degenerative diseases of the central nervous system [18] and the retina [19,20,21], as well as in the development and progression of several ocular diseases [22,23,24]. Obesity is a complex condition associated with an increase in a number of inflammatory markers, resulting in chronic low-grade inflammation [25]. Dysbiosis of the gut microbiome was linked to low-grade inflammation in obese individuals [26,27]. In addition, several ocular illnesses are related to neuroinflammation [28,29], and accumulating evidence suggests neuroinflammation is a crucial component in glaucoma [30,31], but the exact functions remain unknown. Maintaining intestinal homeostasis and inhibiting inflammatory processes requires dynamic interactions between the gut microbiome and the host’s immune system. Gut dysbiosis can dysregulate immune responses by causing intestinal barrier malfunction, resulting in the translocation of bacteria through the epithelial barrier, and causing systemic inflammation predominantly through the generation of proinflammatory cytokines and modifications of lymphocyte populations. This inflammation may lead to tissue degeneration, hence promoting the emergence of numerous illnesses, including eye diseases. As a result of bacterial translocation, bacteria and their metabolites are delivered via the bloodstream or mesenteric lymphatic vessels to ocular regions at the level of the retina and optic nerve, causing tissue degeneration and neuroinflammation. There is now preliminary evidence for the existence of a brain and intraocular microbiome [32,33]. The dysbiosis of the gut could influence the resident brain–ocular microbiome ecosystem which, in turn, could exacerbate the local inflammation. All these processes, finally, could lead to the death of RGC and neurodegeneration. The purpose of this literature review is to explore the recent evidence on the role of gut microbiome dysbiosis and related inflammation as common mechanisms underlying obesity and glaucoma.

## 2. Glaucoma: Intraocular Pressure/Ocular Hypertension and Relationship with Obesity

Although the etiopathogenesis of glaucoma is not well understood, the primary risk factor for glaucomatous optic neuropathy is elevated intraocular pressure (IOP > 21 mmHg), which seems to be linked to the death of retinal ganglion cells (RGCs) in both acute closed-angle glaucoma, in which there is a sudden increase in IOP, and primary open-angle glaucoma, in which the increase in IOP occurs more gradually over the years [11,12]. Elevated IOP over time causes optic nerve damage and vision loss [11,12]. Treatment with medication or surgery to reduce IOP appears to slow the progression of the disease [34]. The IOP in glaucoma patients is caused by a combination of factors, including increased resistance to aqueous drainage through the trabecular meshwork (primary open-angle glaucoma) and iris obstruction of the drainage pathway (primary closed-angle glaucoma) [35]. While IOP is reduced, progressive damage may still be present in certain people with glaucoma. Increased IOP is only one factor in the development of glaucoma; other factors include neuroinflammation [30,31], decreased ocular blood flow [36,37], ocular vascular dysregulation [36,38], and changes in systemic blood pressure [39,40]. The elevation of IOP, along with other vascular abnormalities, such as hypertension and atherosclerosis, is linked to obesity [9,41]. Glaucoma may be affected by metabolic health status and obesity [9,41,42,43,44,45]. In several clinical studies, it has been shown that a high body mass index (BMI) correlates with decreased choroidal perfusion, decreased ocular blood flow, higher orbital fat, and higher IOP; all these factors may negatively contribute to glaucoma development [10,46,47,48,49]. The majority of epidemiological studies have focused on the connection between obesity and IOP, or ocular hypertension. Various studies have revealed an independent cross-sectional connection between obesity and ocular hypertension using population-based data [47,50,51,52,53,54,55,56,57,58,59,60]. A high BMI was found to be a significant risk factor for glaucoma in the Gangnam Eye Study [61], and a link between high BMI and high IOP was found in the 2008–2010 Korea National Health and Nutrition Examination Survey [62]. In a recent and large clinical study of over 40,000 Korean subjects, it was seen that the obesity index is the best indicator for further increases in IOP in the ocular hypertension group [52]. Obese adults exhibit higher open-angle glaucoma risk, according to a Taiwanese study using two databases and matched cohorts [63]. In another study on the Korean population, Jung et al. [9] came to a comparable result that obesity and metabolic health status are strongly related to increased open-angle glaucoma (OAG) risk. A similar causal relationship between obesity and OAG was described by Lin et al. [50] in a recent two-sample Mendelian randomized investigation carried out in China. In prospective population research that included people from Spain and Portugal, it was recently discovered that half of those with OAG and ocular hypertension were overweight or obese [64]. A recent cross-sectional study in Italian children showed that high IOP affects 12.5% of 8-year-old schoolchildren and appears to be associated with high blood pressure related to a high BMI [65]. The conventional view assumes that the higher red cell aggregation, hematocrit, and hemoglobin levels in fat people cause their blood to be more viscous [66]. An increase in IOP may occur as a result of the increased resistance to the outflow of aqueous humor from the eye caused by the afflux reduction [67]. Vascular dysregulation and vasospasms can be caused by obesity and atherosclerosis of the arteries; they participate in blood flow distribution to the optic nerve head, retina, and choroid [68,69]. A reduction in blood flow to the optic nerve can render the nerves more susceptible to harm from an increase in IOP [70,71].

## 3. Microbiome Dysbiosis at the Intersection of Obesity and Glaucoma

### 3.1. Role of Dysbiosis in Obesity Development

#### 3.1.1. Gut Dysbiosis

*Firmicutes*, *Bacteroidetes*, *Actinobacteria*, *Proteobacteria*, *Fusobacteria*, and *Verrucomicrobia* are the most common microbial phyla found in the gut [72,73]. These microorganisms play an essential role in nutrient absorption, energy regulation, and the health of the mucosal barrier [74,75,76]. Dysbiosis in the gut microbiome has been linked to metabolic diseases such as obesity [14,15] and chronic liver diseases [15,16,17]. Changes in the microbiome may influence energy balance and dysregulate immunological responses in obese individuals by inducing intestinal barrier dysfunction, leading to the translocation of bacteria across the epithelial barrier and triggering systemic inflammation [14,15]. Initially, changes in the *Firmicutes* and *Bacteroidetes* phyla were observed in obese animals [77,78,79]. The first studies with obese mouse models showed a decrease in *Bacteroidetes* and an increase in *Firmicutes* [77]. Other studies in leptin-deficient mice corroborate the higher proportion of *Firmicutes* to *Bacteroidetes* in the gut microbiome [78,79]. One study found no change in this variable in obese animals, while another found a decreased *Firmicutes*/*Bacteroidetes* ratio [80]. Several phyla of gut microbes have been linked to an altered ratio in obese individuals. There is substantial evidence that the human gut microbiome differs significantly between people who are obese and healthy controls [77,81,82,83]. There is some consensus that people with obesity and type 2 diabetes have higher levels of gut *Firmicutes* and lower levels of *Bacteroidetes* [84]. The introduction of solid food and infant formula has a significant impact on the composition of the gut microbiota in early life [85,86,87]. Obesity in infants was associated with a predominance of *Firmicutes* and a subsequent dominance of *Bacteroidetes* in the infant’s gut microbiome [87], a finding that is consistent with studies on adults [75,88,89,90]. Some specific bacteria species are linked to a higher susceptibility to developing obesity. *Blautia wexleri*, *Clostridium bolteae*, *Flavonifractor plautii*, and *Ruminococcus obeum* are more common species found in obese patients than in nonobese patients [88]. Obese people may also have an increase in the bacterial species *Eubacterium rectale*, *Clostridium coccoides*, *Lactobacillus reuteri*, *Akkermania muciniphila*, *Clostridium histolyticum*, and *Staphylococcus aureus* [89]. In another study, *Firmicutes*, *Fusobacteria*, *Proteobacteria*, *Mollicutes*, and *Lactobacillus* reuteri were found in higher numbers in obese people, while *Verrucomicrobia* (*Akkermansia muciniphila*), *Faecalibacterium prausnitzii*, *Bacteroidetes*, *Methanobrevibacter smithii*, and *Lactobacillus plantarum* and *paracasei* were found in lower numbers [91]. Palmas et al. [92] confirmed that the number of certain bacterial taxa from the family *Enterobacteriaceae*, which are known to have endotoxic activity, was higher in the obese group than in the normal weight control group. A higher ratio of *Firmicutes* to *Bacteroidetes* has been linked to obesity, according to the majority of clinical studies, but other studies have failed to find a connection between the two and even found evidence of the contrary. However, there may be methodological differences attributed to these divergent findings. Schwiertz et al. [93] found that the proportion of the genus *Bacteroides* was found to be higher in overweight volunteers than in lean and obese volunteers, and the ratio of *Firmicutes* to *Bacteroidetes* shifted in favor of *Bacteroidetes* in these groups. Weight loss did not alter the ratio of *Bacteroides* to *Firmicutes* in the human stomach, as found by Duncan et al. [94]. Furthermore, another study found no statistically significant differences in the ratio of *Firmicutes* to *Bacteroidetes* between obese and normal-weight adults [95] or children [96]. In addition, a recent meta-analysis has shown that neither the proportion of *Firmicutes* nor *Bacteroidetes* nor their individual abundances differentiates healthy human microbiomes from those of people who are overweight or obese [97]. At any rate, the abundance of the phyla *Firmicutes* and *Bacteroidetes* may vary greatly from one population to the next, but this variation may be explained by other factors, such as differences in diet, exercise, food additives and toxins, antibiotic use, and overall level of physical exertion, as well as methodological differences between studies. Gut microbiome dysbiosis could contribute to the proliferation of pathogenic bacteria and vice versa. For example, mice fed with a low-fiber diet experienced more degradation of the mucus layer by *Akkermansia*, and consequently increasing susceptibility to the gut pathogens *Citrobacter rodentium*, *Clostridioides difficile*, and *Salmonella typhimurium* [98,99]. Studies have shown that individuals infected with *H. pylori* tend to be overweight [100] and have a higher BMI compared to the general population, although this association was not detected in another study [100]. Conversely, overweight people were at greater risk of contracting *H. pylori* [101]. According to two studies conducted on the Chinese population, the prevalence of *H. pylori* infection was higher in obese people than in nonobese people [102,103]. Another study found that subjects with *H. pylori* infection and those aged less than 50 years have an increased risk of being obese (BMI ≥ 30) compared to those without this type of infection. However, other researchers have found conflicting results when examining the link between *H. pylori* infection and the onset of obesity [104,105,106,107,108]. Nevertheless, a recent meta-analysis found that *H. pylori* infection is associated with an increased risk of developing obesity [101].

#### 3.1.2. Oral Dysbiosis

Even though gut bacteria are the primary focus of several investigations, the mouth cavity is a possible seeding ground for all gastrointestinal bacteria [109]. The oral microbiome is a part of the gut microbiome family and several oral species are found in the intestine [110]. There is increasing data that show a link between some oral bacterial taxa and weight increase and obesity [111,112]. While some research has found no association between BMI and oral microbiota composition [113,114], other research has found significant differences [111,115,116]. So, there may be a correlation between periodontal disease and excess weight. The prevalence and abundance of periodontal infections including *Tannerella forsythia* and *Selenomonas noxia* have been reported to rise in obese adults [117]. Periodontal disease and abscesses are dominated by *Prevotella* species, and they are frequently linked to mucosal inflammation [118]. Similar to what we found in saliva, *Prevotella* in the stomach has been demonstrated to be inversely related to a child’s BMI and fat mass [119]. Other investigations of adolescents and adults have linked *Prevotella* to aging and proinflammatory cytokines, which is consistent with observations that obesity is related to low-grade inflammation [120]. Raju et al. [121] found, in a study of 483 boys and 417 girls, differences in bacterial diversity and abundance that correlated not only with BMI but also with gender. There are multiple processes by which weight gain may affect the oral microbiota or vice versa. As with gut *Firmicutes*, many hypothesize that oral bacteria could contribute to systemic metabolic changes. Specified oral taxa may contribute to shifting energy expenditure by aiding insulin resistance by raising tumor necrosis factor-alpha (TNF-α) and lipopolysaccharide levels. Additionally, the oral microbiome may contribute to taste perception and hunger regulation [116,122].

### 3.2. Role of Microbiome Dysbiosis in Glaucoma Development

#### 3.2.1. Gut Dysbiosis

As aforementioned, several studies have reported that obesity has a positive correlation with increased IOP and an increased risk of developing glaucoma [46,49,51,52,65,123,124]. In young adults in particular, obesity could be a potential risk factor for glaucoma [63]. On the other hand, the gut microbiome is now being recognized as a potential environmental factor in the development of multiple neurodegenerative diseases and also ocular disorders [125,126,127,128]. The gut microbiome has been shown to influence both the blood–brain barrier (BBB) and brain function [129,130]. Increased intestinal permeability caused by gut dysbiosis allows for the accumulation of microbiome and metabolites in the central nervous system [131,132]. Glaucoma is a multifactorial neurodegenerative disease characterized by the death of retinal ganglion cells (RGCs). Biological mechanisms similar to those proposed for glaucoma and other neurodegenerative diseases involve the loss of particular nerves and the deposition of protein aggregates in specific anatomical areas [133,134,135,136]. Given that glaucoma and other neurodegenerative diseases have similar immune and neurodegenerative factors, the progressive neurodegeneration in glaucoma could be caused by microbial communication between the gut and the eye. As with studies on obese individuals, some studies have investigated the association between specific bacterial phyla present in the gut and the risk of developing several eye diseases; for example, dry eye, autoimmune uveitis, and age-related macular degeneration have all been linked to an increased Firmicutes/Bacteroidetes ratio as found in animal models [125,126,127]. However, there are few studies that have investigated the association between gut microbiome dysbiosis and glaucoma. For example, DBA/2J mice, an animal model of glaucoma that normally develops elevated IOP and glaucoma by 6–8 months of age, do not show any signs of glaucomatous neural degeneration at 12 months of age when raised in a germ-free environment [137] while DBA/2J mice that have been maintained in a specific pathogen-free environment have been shown to suffer from a progressive loss of RGCs and axons, with percentages of 25% and 50%, respectively, at 8–10 months and 12 months of age [137]. In the glaucomatous rat model, the Firmicutes/Bacteroidetes ratio, the Verrucomicrobia phylum, and some bacterial genera (Romboutsia, Akkermansia) were drastically elevated compared to the control, and this was inversely correlated with RGCs [128].

When comparing patients with primary open-angle glaucoma (POAG) to healthy controls, Gong et al. [138] discovered that the microbial composition of their guts varied: Prevotellaceae, Enterobacteriaceae, and Escherichia coli increased in abundance in POAG patients compared to healthy controls, whereas Megamonas and Bacteroides plebeius decreased significantly. Intriguingly, mean visual acuity and the visual field mean defect (VF-MD) had a negative correlation with Blautia, while the visual field mean defect (VF-MD), which reflects axonal loss within the optic nerve, had a positive correlation with Streptococcus [138]. In addition, they found that the change in gut microbial species led to a variation in circulating metabolites in POAG and linked these taxa to specific metabolites that may play a role in glaucoma pathogenesis [138].

A clinical study involving POAG patients compared with the healthy controls found that two variants of mitochondrial DNA (m.15784T > C and m.16390G > A) in the DNA pools are associated with the composition of the gut microbiota [139]. Particularly, the variant m.15784T > C was associated with *Firmicutes* members, while the variant m.16390G > A was linked with *Proteobacteria* members [140]. In POAG, increased mtDNA deletion correlates with fewer mitochondria per cell and increased cell death [141]. Patients with POAG show a range of mitochondrial abnormalities, and oxidative stress may cause glaucoma-related apoptosis by damaging mitochondria [142]. Metabolites produced by intestinal flora are the mediators of gut microbiota–host interactions. Blood and eye fluid samples from glaucomatous patients have been analyzed in metabolic studies, and unique metabolic phenotypes have been identified [138,143,144,145,146,147,148,149]. As for dysbiosis in obesity, a possible link between glaucoma and *Helicobacter pylori* was also highlighted by some research groups [14,101,106]. Kountouras et al. [150] in the year 2000 discovered a correlation between glaucoma and the gut microbiome by finding that 88% of 281 glaucoma patients had a gastric *H. pylori* infection compared to 47% of controls. The results of subsequent investigations that included serology and/or breath testing were inconclusive [151], but two meta-analyses generally indicated evidence of a connection between active *H. pylori* infection and POAG [152,153]. It has been theorized that *H. pylori* infection exacerbates glaucoma by increasing systemic inflammation, vasoactive and reactive oxygen species [154], and antibody-dependent responses to cross-reactive ocular antigens [155]. Reactive oxygen species and inflammatory cytokines migrate from the gastric mucosa to the optic disc or trabecular meshwork and *H. pylori* IgG antibodies may cross-react with ocular tissues [156]. The eradication of *H. pylori* infection has been shown to reduce IOP [157] and enhance visual fields [158] in limited studies of POAG patients [159]. Further research on gut microbiota in POAG has interpreted the correlation with *H. pylori* infection as evidence that intestinal dysbiosis is a risk factor for both disorders [160], although it is still unknown if the two diseases are causally linked or if the observed association is due to shared susceptibility.

#### 3.2.2. Oral Dysbiosis

A greater number of oral bacterial organisms (e.g., Streptococci) and poorer oral health (fewer teeth) were found in patients with glaucoma compared to healthy controls in studies that investigated a possible relationship between the oral microbiome and glaucoma [161,162]. Pasquale et al. [163], using data from the health professionals’ follow up, found that losing teeth in the two years before a glaucoma diagnosis was related to a 1.45-fold greater risk of POAG. If tooth loss was followed by periodontal disease with bone loss during the same period, the multivariate relative risk (MVRR) increased to 1.85 [163]. Therefore, oral dysbiosis of the microbiome may initiate and/or worsen glaucomatous pathology, as shown by clinical data [163]. Yoon et al. [164] found a significant oral depletion of *Lactococcus*, while *Faecalibacterium* was enriched in glaucomatous patients compared to healthy controls.

#### 3.2.3. Ocular Dysbiosis

The vitreous and aqueous humors contain a variety of organic and inorganic compounds that provide an ideal habitat for microbial growth [165]. For example, *Propionibacterium* acnes was discovered in the granuloma of the retina in patients with ocular sarcoidosis, suggesting that *P. acnes* may be linked to sarcoid uveitis [166,167]. Despite the fact that *H. pylori* are an obligatory colonizer of stomach mucosa [168], one investigation revealed *H. pylori* coccoid forms in trabecular and iris specimens from POAG patients [156]. In the year 2021, the existence of a resident brain microbiome was hypothesized [32]; moreover, there is preliminary evidence of intraocular microbiome and disease-specific microbial signatures in eyes affected by senile cataracts, age-related macular degeneration, and glaucoma [33]. Although the presence of intraocular microbiota in healthy eyes has yet to be confirmed by others, the findings of Deng et al. [33] suggest that the commensal microbiome is a part of the retinal ecosystem and may alter the intraocular microenvironment and regulate the retinal immune response directly in retinal degeneration. Disease-specific alterations in the composition of the microbiome found within the eye hint at a possible selection mechanism operating along the gut–eye axis [33]. The interaction between neurons and immune cells, as well as the retinal pigment epithelium and immune cells, is crucial to the suppression of the immune response in the retina [169] and also in glaucoma disease [170,171]. Hence, neuronal and retinal pigment epithelium degeneration may alter the immune suppressive property, resulting in new ocular or brain microbiota.

**Figure 1:**
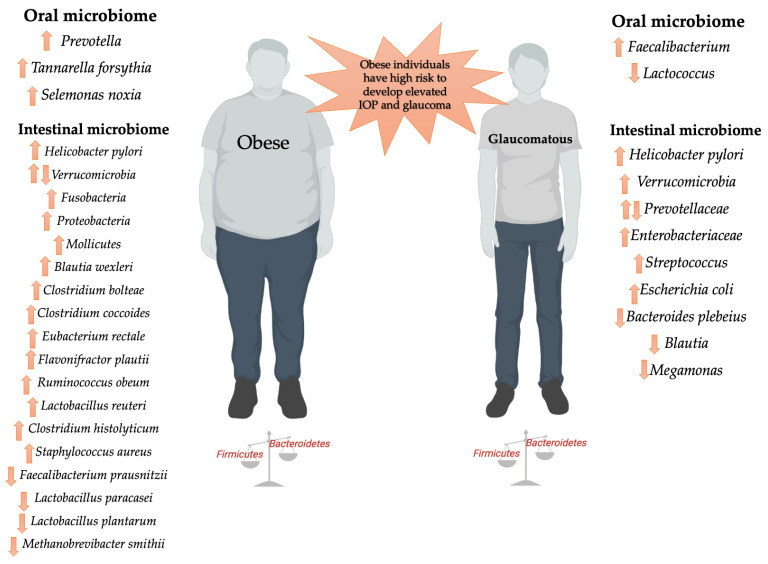
Dysbiosis in obese and glaucomatous individuals is characterized by a decrease in total bacterial diversity and richness, as well as, in general, by a shift toward a community composed of more *Firmicutes* and fewer *Bacteroidetes*. Created with BioRender.com (accessed on 23 December 2022) and modified with Microsoft PowerPoint.

Together, the aforementioned studies provide support for the involvement of an altered microbiome (in general, a community composed of more *Firmicutes* and fewer *Bacteroidetes*) in the pathogenesis of obesity and glaucoma (Figure 1). However, additional work is required to further elucidate this association.

### 3.3. Inflammation Mediated by Microbiome Dysbiosis in Obesity

Changes in the inflammatory profile, including chronic low-grade systemic inflammation, occur in conjunction with obesity and fat storage [172,173,174]. Inflammatory mechanisms contribute to the formation and progression of insulin resistance [175,176]. Adipose tissue inflammation may begin with the production of inflammatory mediators, which then leads to macrophage infiltration, which in turn exacerbates inflammation [177]. Both resident and invading macrophages in adipose tissue can become activated, starting off a chain reaction that ultimately results in the production of many proinflammatory cytokines [178].

Several pieces of evidence showed that obese individuals have a high level of endotoxins in the blood, which is related to higher proinflammatory cytokines such as TNF-α and IL-6 in adipocytes [179]. Compared to lean individuals, the levels of LPS were shown to be 20% higher in individuals with obesity or glucose intolerance and 125% higher in those with type 2 diabetes [180]. A study in vivo showed that injecting LPS into genetically identical male mice for four weeks caused a weight increase equivalent to that observed in animals fed a high-fat diet [181].

Gut microbiome dysbiosis has been proposed as a pathogenic mechanism at the base of metabolic endotoxemia and related inflammation in obesity disease [181]. Intestinal homeostasis and the suppression of inflammation depend on the dynamic interactions between the gut microbiome and the host immune system [182]. The intestinal epithelium serves as a biochemical and physiological barrier between the host and foreign antigens such as those found in food, commensals, infections, and toxins. The gut microbiome plays a crucial role in inflammatory signaling by producing a wide variety of metabolites, including LPS, which mediate communication between the gut epithelium and immune cells [26]. The mutualistic bacteria of the gut microbiome protect the host from pathogens by using a variety of mechanisms, such as nutrient competition, altering environmental conditions, modulating immune cell maturation (immune-mediated resistance), and producing metabolites with growth-limiting or bactericidal effects [183]. Nonetheless, commensal bacteria can turn pathogenic after mucosal translocation or under certain conditions (such as immunodeficiency) [184].

The altered gut microbiome leads to an increase in gut permeability and systemic levels of bacterial products such as LPS [185]. LPS generated from the outer cell membrane of gram-negative bacteria is believed to trigger the inflammatory processes linked to the formation of obesity and insulin resistance [185,186]. LPS can traverse the gastrointestinal mucosa through leaky intestinal tight junctions or by infiltrating chylomicrons, the lipoproteins responsible for the absorption of dietary triglycerides and cholesterol from the intestine to the plasma [186,187]. Excessive fat consumption causes a rise in chylomicrons in the colon during the postprandial period, which promotes LPS penetration into the circulation [188]. In patients with type 2 diabetes, impaired lipoprotein metabolism reduces LPS catabolism and may exacerbate endotoxemia-related inflammation [189]. When LPS reaches the systemic circulation, it infiltrates organs such as the liver and adipose tissue, causing an innate immune response [185,186]. Specifically, LPS binds the plasma LPS-binding protein, which activates the macrophage plasma-membrane-located CD14 receptor protein [186,190]. This complex, in turn, binds the toll-like receptor 4 (TLR4), a pattern recognition receptor (PRR), on the surface of macrophages, which activates the production of genes encoding various inflammatory effectors, including nuclear factor B (NF-κB) and activator protein 1 (AP-1) [190,191]. NF-κB is recruited to the nucleus, where it stimulates the transcription of proinflammatory cytokines such as interleukin-6 (IL-6), TNF-α, interleukin-1 beta (IL-1β), inducible nitric oxide synthase (iNOS), and cyclooxygenase 2. (COX-2) [192]. LPS also regulates the nucleotide oligomerization domain (NOD)-like receptors found on macrophages and dendritic cells, which work in conjunction with TLRs to induce NF-κB. Moreover, LPS recruits other effector molecules, such as the nucleotide-binding domain leucine-rich repeat-containing (NLR) protein, adaptor protein ASC, and caspase-1, which are components of the inflammasome, a multiprotein oligomer that activates the innate immune system [193].

As aforementioned, endotoxin-induced inflammation is highly associated with the immunological response mediated by TLRs. TLRs have been linked to the persistent inflammation that characterizes metabolic syndrome and obesity [172,174]. TLR4 is one of the best-studied TLR members, which has been shown to identify both LPS and heat shock proteins (HSP), triggering immunological responses to these external and endogenous ligands, respectively [194,195,196]. TLR4 is expressed by adipocytes, and its activation leads to the production of proinflammatory cytokines and an intense immune response, both of which may play a role in the etiology of obesity [197]. Improved TLR4 mRNA expression was seen in the adipose tissue of obese db/db mice, suggesting a possible role for TLR4 signaling in both obesity and inflammation [198]. Conversely, the knockout of TLR4 inhibits the development of obesity-related illnesses [199,200,201] and lowers weight gain in young mice fed a high-fat diet [202]. Inflammation and insulin resistance, caused by obesity, is reduced in TLR4-/- mice by preventing insulin signal transduction and nitric oxide generation [203]. Moreover, animals lacking TLR4 were protected against insulin resistance generated by a high-fat diet [204]. In an ex vivo study, Renovato-Martins et al. [205] demonstrated that the conditioned medium derived from obese adipose tissue induces inflammation in preadipocytes via increased TLR4 signaling and ROS production, thereby creating a paracrine loop that promotes the differentiation of preadipocytes into adipocytes with a proinflammatory profile. High levels of circulating free fatty acids (FFAs) are associated with obesity and have been demonstrated to induce insulin resistance via many proinflammatory pathways [174,206,207,208]. The levels of FFAs in the blood of obese patients were substantially higher than those of lean subjects [209]. Increased TLR4 signaling in adipose tissue, liver, and macrophages was reported in mice by Shi et al. [204] in response to the elevation of FFA levels. The stimulation of TLR4 expression on macrophages, adipocytes, and adipose tissue by FFAs leads to an increase in the release of several inflammatory mediators [204,210,211]. Research in humans confirms the elevated TLR activation, seen in animal studies, in both adipose tissue and peripheral monocytes from obese individuals. Muscle samples from obese patients showed higher levels of TLR4 expression and NF-κB activity, leading to higher levels of proinflammatory IL-6 release, compared to biopsies from lean subjects [209]. Omental fat tissue has significantly higher mRNA expression of TLR1, TLR2, TLR4, and TLR6 [212]. Ahmad et al. [213] discovered significantly higher expression levels of TLR2 and TLR4 in peripheral blood mononuclear cells and subcutaneous adipose tissue of obese and overweight people in comparison to lean controls. When comparing peripheral monocytes from obese and healthy controls, Mraz et al. [214] found significantly higher levels of TLR4 expression. Similarly, Vitseva et al. [210] found an elevated level of TLR4 mRNA expression in adipocytes isolated from human subcutaneous abdominal fat from obese people, together with elevated NF-κB activity and the release of IL-6 and TNF-α. Instead, Catalán et al. [215] found higher TLR4 mRNA expression in visceral adipose tissue, but not subcutaneous adipose tissue, in normoglycemic obese people compared to lean controls. Flow cytometry analysis showed that TNF-α production was significantly increased in obese groups compared to nonobese patients. Nonetheless, the analysis did not reveal an increase in TLR2 or TLR4 expression [216]. In addition, two studies found that some TLR4 polymorphism is linked to a higher probability of developing obesity and metabolic disorders, and this is proof that supports the key role of TLR4 in these diseases [217,218]. Sharif et al. [219] found that the TLR4 D299G/T399I haplotype polymorphism elevates the risk of insulin resistance by elevating TLR4 protein expression in obese subjects.

### 3.4. Inflammation Mediated by Microbiome Dysbiosis in Glaucoma Pathogenesis

Human retinal ganglion cells, retinal nerve fiber layer thickness, and choroidal thickness can all be significantly altered by obesity [220,221,222]. Several pieces of evidence showed that glaucomatous eyes have increased protein levels of proinflammatory cytokines, macrophage infiltration of the optic nerve [223,224], and intense inflammatory staining in the optic nerve head, which can damage the optic axon [225,226]. Patients with glaucoma not only have aberrant circulating antibodies but also altered T-cell repertoires [227]. TNF-α is upregulated in glaucoma [228], and the recruitment of immune cells in response to active TNF-α receptor 1 causes inflammation, the loss of oligodendrocytes, the activation of enzymes that induce oxidative stress, and ultimately the death of RGCs [229]. The complement components, autoantibodies, and other inflammatory markers have all been found to be elevated in the blood, aqueous humor, and vitreous of glaucomatous patients [230,231,232,233]. Hence, the abnormal inflammatory findings in glaucoma are consistent with the hypothesis that the inflammatory balance is dysregulated toward a proinflammatory phenotype [229].

Obesity is correlated with gut dysbiosis and an increased risk of developing glaucoma [46,49,51,52,63,65,123,124]. On the other hand, gut microbiome dysbiosis appears to play a crucial role in the development of several ocular diseases [234,235,236] and glaucoma [137]. Dysbiosis and the consequent impairment of gut permeability result in an increase in gut-derived toxins in the systemic circulation, producing metabolic endotoxemia. The translocation of microorganisms and metabolites from the gut into the circulatory system and other tissues has been linked to the onset of autoimmune and neurodegenerative disorders [237,238]. Retinal degenerative diseases may be the outcome of gut microbial dysbiosis, which causes a low-grade systemic inflammation [239,240]. By modifying the systemic immune system and/or influencing the ocular microenvironment, microbial dysbiosis could have a role in the onset and progression of inflammatory eye disorders [20,241], including glaucoma [242].

In vivo studies in animal models of glaucoma found that the blood–retina barrier (BRB) is altered [243,244] and that monocytes can actively extravasate across the leaked BRB and passively enter the eye [245,246]. Additionally, in humans, the significant infiltration of monocytes into the optic nerve head has been demonstrated in glaucoma [224].

Gut microbiome dysbiosis (including that of the oral cavity) could induce a state of endotoxemia where higher access to metabolites and nonbeneficial bacteria from the systemic environment could, in turn, alter the brain, retinal, and ocular barriers [247,248,249,250]. The alteration of these barriers, in turn, could allow an increased influx of inflammatory metabolites and bacteria into the ocular regions at the retinal and optic nerve level, which could contribute to local inflammation (neuroinflammation) which has a prominent role in glaucoma pathogenesis [31]. The gut microbiome has been shown to influence both the blood–brain barrier (BBB) and brain function [129,130]. Increased intestinal permeability caused by gut dysbiosis allows microbes and metabolites to accumulate in the central nervous system [131,132]. The author Link [32], in his work, postulated the existence of a resident brain microbiome, and Deng et al. [33] found evidence of intraocular microbiota and ocular-disease-specific microbial signatures in eyes affected by senile cataracts, age-related macular degeneration, and glaucoma, in which the alteration of the blood–ocular barrier (BOB) seems to be involved. In response to endotoxemia, the brain–ocular microbiome ecosystem may be altered, which would exacerbate the local inflammation. Moreover, the ocular microbiome could modulate the retinal immune response directly by activating TLRs or indirectly by releasing various metabolites in the degenerating retina [170,171].

Inflammation caused by endotoxins is closely associated with the immunological response mediated by TLRs. TLR expression was upregulated in both retinal microglia and astrocytes [251] and has been linked to the pathophysiology of the degeneration of RGCs in glaucoma and other ocular disorders [252,253,254,255,256]. Results from in vivo investigations suggested that blocking TLR4 signaling could be a useful strategy for treating glaucoma [257,258,259,260,261]. Zinkernagel et al. [262] showed that a peripheral injection of bacterial LPS induced axonal degeneration and neuronal reduction in two distinct glaucoma animal models. This result was likely attributable to TLR4 overexpression, complement activation, and injury to the retina and optic nerve microglia; consequently, the bacterial activation of axonal microglia promotes neurodegeneration [262]. TLR4, which is expressed by RGCs and other retinal cells, responds to a variety of endogenous ligands, which can be found in the AH and vitreous humor (VH) of individuals with retinal ischemia disorders and glaucoma [263,264,265,266]. TLR4 suppression strategies were tested in animal models of retinal damage to corroborate the deleterious roles of TLR4 in RGC mortality. RGC survival is improved in a model of optic nerve trauma by inhibiting TLR4 with inhibitors or knockouts [257,261]. Further, silencing TLR4 inhibits the proinflammatory response triggered by amyloid through the inhibition of NF-κB activation in RGCs [267]. TLR activation can also occur via self-components, as seen in autoimmune diseases [268,269]. Heat shock proteins (HSPs) are a type of nonpathogenic ligand that TLRs can recognize [268,269]. An analysis of TLR presence in human glaucoma donor eyes also revealed that HSPs and oxidative stress can stimulate immune activity in rat retinal microglia and astrocytes in vitro via glial TLR signaling [270,271]. Increased TLR expression was observed after incubating cells with HSP60 and -70 and then subjecting them to radical stress [272,273]. TLR2, TLR3, and TLR4 were detected by immunohistochemistry on both rat retinal microglia and astrocytes in vitro, with TLR3 being more abundant on astrocytes and TLR2 and TLR4 being more abundant on microglial cells [272,273]. Mass spectrometry showed an upregulation of TLR4 in the retinae of glaucomatous donor eyes [274]. Additionally, proteomic and immunohistochemical analyses revealed an upregulation of TLRs in the glaucomatous human retina [271].

There are several single-nucleotide polymorphisms that have been linked to an increased risk of glaucoma because of their potential to affect inflammation [275]. As for obesity, several TLR4 polymorphisms are linked to a higher probability of developing glaucoma, and this is proof that supports the key role of TLR4 in the pathogenesis of this disease. The relationship between TLR4 polymorphisms and glaucoma has been the subject of numerous case-control investigations over the past few decades, but the results have been conflicting in some cases [154,276,277,278,279,280,281,282,283]. Suh et al. [283] showed that TLR4 polymorphisms are not necessarily linked to normal tension glaucoma (NTG) pathogenesis in the South Korean population. Takano et al. [282] found that some variations in the TLR4 gene were linked to an increased risk of POAG and NTG. Two recently discovered variants of TLR4, rs4986790 A/G and rs4986791 C/T, were found to significantly elevate the risk of POAG in a Mexican population [277]. However, Mousa et al. [280] did not find any link between rs4986791 C/T and POAG in a study on the Saudi population with a much smaller sample size. Evidence suggests that these functional polymorphisms promote apoptosis in hepatic stellate cells by decreasing Bcl-2 [284,285], although the roles of these mutations are controversially discussed for various diseases. The polymorphisms rs4986790 A/G and rs4986791 C/T were therefore proposed to promote RGC apoptosis [277]. Rs4986790 A/G and rs4986791 C/T polymorphisms do not interfere with the LPS binding property of TLR4, as shown by a structural protein study using crystallography [286]. The mutations may affect TLR4’s response to damage-associated molecular pattern molecules (DAMPs) [264,265,266,287]. In a Japanese population, POAG was linked to TLR4 polymorphisms rs7037117 [282]. Of interest, rs7037117 also showed a strong correlation to NTG [154].

**Figure 2:**
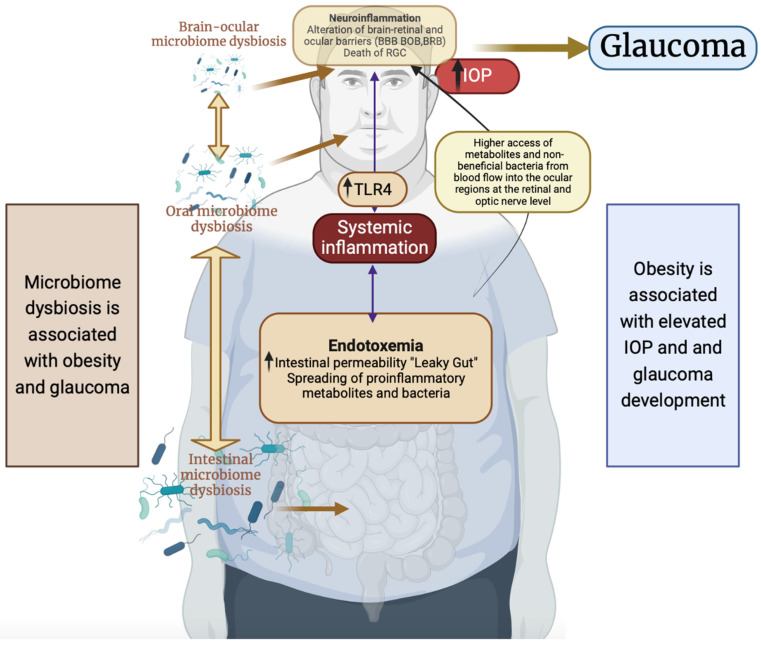
Microbiome dysbiosis and related inflammation: pathological mechanisms underlying obesity and glaucoma. BBB: blood–brain barrier; BRB: blood–retinal barrier; BOB: blood–ocular barrier; IOP: intraocular pressure; RGC: retinal ganglion cells; TLR4: toll-like receptor 4. Created with BioRender.com (accessed on 23 December 2022).

In summary (Figure 2), obesity is correlated with gut dysbiosis, and the obese individual has a high risk of developing elevated IOP and glaucoma. In a gut dysbiotic scenario, the systemic spread of proinflammatory metabolites and bacteria through the leaky gut could induce systemic inflammation, where TLR4 activation plays a prominent role, and could alter the retinal and ocular barriers (BBB, BRB, and BOB), resulting in increased access to metabolites and nonbeneficial bacteria in the ocular regions at the retinal and optic nerve level. This event, in turn, could contribute to local inflammation (neuroinflammation). Moreover, the dysbiosis of the gut microbiome could influence the resident brain–ocular microbiome, which, in turn, could exacerbate the local inflammation. All these processes led to the death of RGC and neurodegeneration.

## 4. Potential Therapies for Obesity and Glaucoma

**Figure 3:**
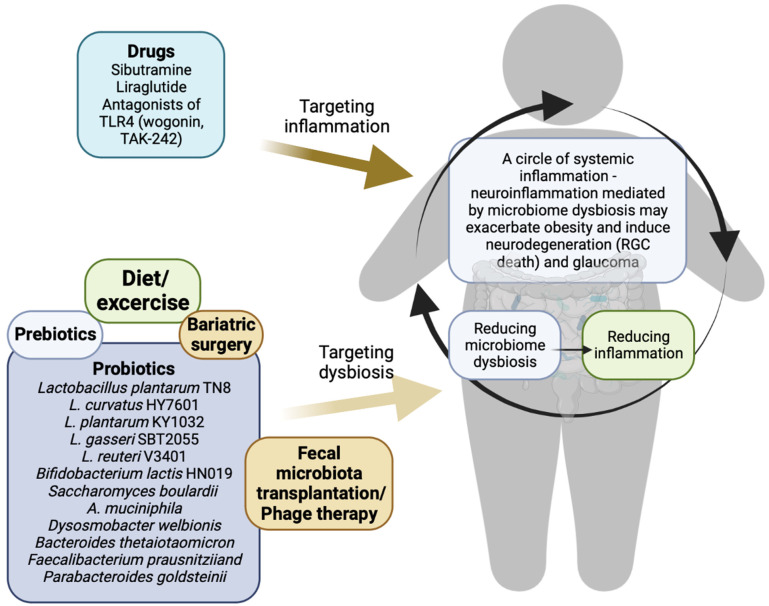
Potential therapies for obesity and glaucoma. Obesity is correlated with gut dysbiosis, and obese individuals have a high risk of developing elevated IOP and glaucoma. Gut dysbiosis could induce systemic inflammation, which is involved in the progression of obesity pathogenesis and various diseases affecting the eye. Promising preventive or therapeutic approaches for both obesity and glaucoma could involve rebalancing microbiome dysbiosis, which is one of the triggers of inflammation in both diseases or directly intervening with anti-inflammatory drugs. RGC: retinal ganglion cell. Created with BioRender.com (accessed on 23 December 2022).

Surely, diet has a fundamental role in the control and prevention of chronic low-grade inflammation in obese individuals [288,289]. Adipose tissue is an endocrine organ that, according to substantial research, plays a crucial role in the homeostasis of immunity [290]. Obesity, a condition of chronic low-grade inflammation, is caused by an excess of nutrients [288,289]. Thus, optimal nutrition plays a crucial role in immunity. The variety, composition, and metabolic activity of the gut microbiome are strongly correlated with dietary habits and nutrient consumption [291]. Additionally, dietary variations alter the composition and activity of the gut microbiome, which may help to reduce obesity [291]. In addition to the diet, exercise is known to stabilize the progression of obesity and alter the composition of the gut microbiota by increasing microbial diversity, which may contribute to weight loss, obesity-related pathologies, and gastrointestinal disorders [292,293]. Moreover, exercise is regarded as an effective nonpharmaceutical therapy for reducing inflammatory signaling pathways [294]. Numerous therapeutic approaches to reduce dysbiosis are now being studied, and some of them concern the use of probiotics (Figure 3). Results from animal research indicate that probiotics can enhance intestinal permeability and the metabolic and inflammatory state [295,296]. *Lactobacillus* and *Bifidobacterium* species are the most widely proposed probiotics for obesity. These species have modest levels of pathogenicity and antibiotic gene resistance [297]. Evidence suggests that lactobacilli can inhibit TNF-α expression, which in turn lowers chronic inflammation and may be useful in the treatment of neurological disease [298]. It has been shown that *L. plantarum* TN8 can reduce proinflammatory IL-12, IFN-γ, and TNF-α levels while increasing anti-inflammatory IL-10 levels in diet-induced obese mice [299]. Adipose tissue proinflammatory cytokines were downregulated, and fat storage was severely impacted by *L. gasseri* SBT2055 supplementation in mice [300]. The effects of *L. curvatus* HY7601 and *L. plantarum* KY1032 were investigated in diet-induced obese mice by Park et al. [301]. Supplementation with either probiotic resulted in less fat being stored and a lower BMI. In addition, the authors found that adipose tissue proinflammatory genes (IL-1, TNF-α, IL6, and monocyte chemotactic protein-1) were suppressed [301]. In obese and type 2 diabetic mice, *Saccharomyces boulardii* administration for one month decreased body weight, hepatic steatosis, fat mass, and inflammation, and altered the composition of the gut microbiota (increasing *Bacteroidetes* and decreasing *Firmicutes*, *Proteobacteria*) [302]. The modulation of cytokines such as IL-6 and TNF-α was observed in *Bifidobacterium lactis* HN019-treated patients with metabolic syndrome in addition to a decrease in weight gain [303]. *L. reuteri* V3401 supplementation in individuals with metabolic syndrome reduced IL-6 and soluble vascular cell adhesion molecule 1 and increased the amount of *Verrucomicrobia* [304]. Results from animal models of obesity showed that treatment with *Akkermansia muciniphila* reduced insulin sensitivity, fat deposition, and weight gain [305]. *A. muciniphila* supplementation reduces hepatic steatosis and intestinal permeability [306]. Amuc 1100, a protein isolated from the outer membrane of *A. muciniphila*, has been shown to interact with TLRs [307]. Similar positive effects to those of *A. muciniphila* were recently discovered in *Dysosmobacter welbionis* [308]. Live *D. welbionis* J115T supplementation reduced weight gain, insulin resistance, and inflammation in white adipose tissue in mice [308]. *Bacteroides thetaiotaomicron* has shown substantial efficacy in preclinical models of inflammatory bowel illness, protecting against weight loss and histological alterations in the colon and inflammatory markers [309]. *Faecalibacterium prausnitziiand* had beneficial effects on intestinal epithelial barrier impairment in a chronic low-grade inflammation murine model [310]. Oral administration of *Parabacteroides goldsteinii* bacteria to mice fed a high-fat diet significantly reduced weight gain and obesity-associated metabolic abnormalities [311].

In addition to probiotics, prebiotics have been proposed as possible ways to reduce inflammation, especially in preclinical models [312], and the impact of antiobesity drugs on microbiota composition and inflammation has been the subject of current research. Additionally, research has shown that bariatric surgery reduces obesity-related comorbidities [313] and chronic low-grade inflammation [314]. Comparing the gut microbiota of obese and lean individuals revealed a larger proportion of *Firmicutes* and a lower proportion of *Bacteroidetes* in obese individuals, whereas the opposite profile was observed in those who underwent one-year diet therapy and a gastric bypass [315]. The malabsorption status following bariatric surgery, changes in the metabolism of bile acids, changes in stomach pH, and changes in the metabolism of hormones result in alterations in the gut microbiota [316].

Several promising therapies, such as fecal microbiome transplantation and phage (bacteriophage) therapies, are currently being investigated. Transplants from lean donors have been shown to increase insulin sensitivity in individuals with metabolic syndrome and decrease persistent low-grade inflammation, according to preliminary trials [317,318]. Using phage (bacteriophages) therapy to target the microbiota of the gastrointestinal tract as a treatment for obesity is a novel approach [319,320,321]. Bacteriophages are viruses that infect bacteria by attaching to distinct binding sites on the cell surface [319,320]. Unlike broad-spectrum antibiotics, each phage exclusively kills a particular type of bacterium [319,320]. This allows for the treatment of obesity by targeting only the harmful bacteria in the stomach while allowing the beneficial bacteria to thrive [321,322]. The novel study by Federici et al. [323] suggests that phage therapy can be utilized to treat not only intestinal bowel diseases but also diseases such as obesity, in which the gut microbiota plays an important role.

Another modality to prevent or possibly treat obesity and glaucoma is to intervene directly with drugs capable of reducing inflammation or regulating proteins/genes that are key components of the proinflammatory mechanisms underlying the two pathologies, such as TLR4. Sibutramine has proven to be more effective at suppressing the expression of flagellum-encoding genes, which have been linked to inflammation [324]. Otherwise, liraglutide has been shown to reduce inflammation by regulating gene expression in both intestinal immune cells and peripheral blood mononuclear cells [325]. Given its involvement in multiple inflammatory and fibrotic processes with different etiologies, TLR4 is an appealing therapeutic target. The lack of TLR4 signaling ameliorated the insulin and glucose signaling abnormalities associated with obesity [204,326]. In addition, TLR4 is recognized as a critical pathogenic molecule in autoimmunity and inflammation [327]. It is interesting to note that numerous different TLR4 antagonists are currently being tested in clinical studies for a wide range of inflammatory illnesses. Anti-TLR4 antibodies are now being tested in phase II studies for the treatment of rheumatoid arthritis, while the TLR4 inhibitor JKB-121 is being tested for the treatment of nonalcoholic steatohepatitis [328]. Studies using acute liver injury models have shown that TLR4 antagonists can decrease TLR4 signaling, the subsequent inflammatory cascade, and liver injury in vivo [329]. Findings by Moser et al. [330] demonstrated a strong protective impact of TLR4 inhibition (TLR4 inhibitor TAK-242) in obesity-mediated outcomes involving the inflammation of microglia. Matsunaga et al. [331] found that the anti-inflammatory activity of the small-molecule TAK-242 is mediated by binding preferentially to the intracellular toll-interleukin-1 receptor (TIR) homology domain of TLR4, blocking the protein’s ability to link to its adapter molecules.

As far as the inflammation in glaucoma is concerned, Xu et al. [259] found that wogonin, a methylated flavone [332], protected RGCs’ survival and reduced neuroinflammation in the retina following crush injury to the optic nerve via the blockade of the TLR4-NF-κB pathways. Hepatic steatosis is significantly related to visceral fat and obesity. As a result of de novo lipogenesis dysregulation, the quantity of FFA molecules rises in hepatic steatosis, triggering oxidative stress [333,334,335]. Fibrosis of the eye, liver, kidney, and skin is characterized by an increase in the expression of extracellular matrix (ECM) proteins and a decrease in ECM degradation, and it is well known that TGF-β2 is a master regulator in these processes [333,334]. Eye, liver, kidney, and skin fibrosis are all characterized by an increase in the expression of extracellular matrix (ECM) proteins and a decrease in ECM degradation, and it is well recognized that the TGF-β is a master regulator in these processes [336]. Patients with POAG have elevated levels of TGF-β2 in their AH [337]. Elevated IOP is brought on by an accumulation of TGF-β2 at the trabecular meshwork (TM), which increases aqueous outflow resistance and promotes ECM synthesis by fibroblasts [338,339,340,341]. In the last few decades, TLR4 signaling for fibroblasts has been hypothesized. Recently, Sharma et al. [342] showed that the modulation of TGFβ2-induced ECM production performed by a selective TLR4 inhibitor decreases elevated IOP and hence might reduce hypertensive glaucoma. These findings suggest a new and realistic method for treating glaucoma.

## 5. Conclusions

Several studies have shown that obese patients have a higher risk of developing elevated intraocular pressure and glaucoma. On the other hand, microbiome dysbiosis has been widely acknowledged to play a role in the etiology of obesity. Research on the link between eye diseases and the gut microbiome has gained attention recently. The role of the microbiome is gradually emerging.

Our review provides evidence that dysbiosis of the gut microbiome and related inflammation are shared pathological mechanisms involved in the development of obesity and glaucoma. Several investigations have demonstrated that gut dysbiosis influences energy balance and dysregulates immunological responses in obese individuals, with a change in intestinal permeability that may promote metabolic endotoxemia. If proinflammatory metabolites and bacteria can enter the bloodstream via a leaky gut, they can trigger systemic inflammation, in which TLR4 activation plays a key role. This, in turn, can disrupt the blood–brain barrier, the blood–retinal barrier, and the blood–ocular barrier, allowing more metabolites and nonbeneficial bacteria to enter the retina and optic nerve. Such events, successively, could contribute to local inflammation (neuroinflammation). Evidence such as this suggests that the alteration in the gut microbiome may have an effect on the microbes normally found in the brain and eye, perhaps enhancing any inflammation that may already be present. All of these factors could contribute to retinal ganglion cell loss and subsequent neurodegeneration in glaucoma disease.

Therefore, according to the data explained in this review, beneficial preventive or therapeutic approaches for both obesity and glaucoma may involve rebalancing microbiome dysbiosis, which is one of the trigger causes of inflammation, or directly intervening with anti-inflammatory drugs that can regulate key components (such as TLR4) of the proinflammatory processes underlying both diseases. Nevertheless, obesity and glaucoma are multifaceted illnesses in which dysbiosis and associated inflammation are not the only pathogenic causes; hence, the link between gut microbiome dysbiosis, obesity, and glaucoma should be the topic of future research.

## Data Availability

Not applicable.
